# Thyroid Function/Antibodies in Sudanese Patients with Preeclampsia

**DOI:** 10.3389/fendo.2015.00087

**Published:** 2015-06-11

**Authors:** Enaam T. Elhaj, Ishag Adam, Ammar Alim, Elhassan M. Elhassan, Mohamed F. Lutfi

**Affiliations:** ^1^Faculty of Applied Medical Science, Gezira University, Medani, Sudan; ^2^Faculty of Medicine, University of Khartoum, Khartoum, Sudan; ^3^Faculty of Medicine, Al-Neelain University, Khartoum, Sudan

**Keywords:** preeclampsia, thyroid-stimulating hormone, thyroxine, tri-iodothyronine, thyroid antibodies, Sudan, thyroid

## Abstract

Preeclampsia is an important cause of maternal and prenatal morbidity and mortality in the developing countries. Changes in thyroid function/antibodies profiles in preeclamptic women are controversial and were never investigated before in Sudan. A case–control study was conducted at Medani Hospital, Sudan, to investigate thyroid function/antibodies in preeclampsia. The sociodemographic, medical history was gathered using questionnaires. Thyroid hormones [thyroid-stimulating hormone (TSH), free tri-iodothyronine (T3), and free thyroxine (T4)] and anti-thyroid peroxidase (anti-TPO) and anti-thyroglobulin (anti-TG) antibodies were measured using ELISA. The three groups [controls, mild, and severe preeclampsia (SP) (55 women in each arm)] were matched in age and parity. While median (interquartile range) of TSH was significantly lower, both free T3 and free T4 levels were significantly higher in women with preeclampsia than in the healthy controls. There was no significant difference in the TSH levels in women with MP and SP. In comparison with women with MP, women with SP had significantly higher levels of free T3 and significantly lower levels of free T4. While anti-TPO antibodies were significantly higher, anti-TG antibodies were significantly lower in women with preeclampsia. Likewise, anti-TPO antibodies were significantly higher and anti-TG antibodies were significantly lower in women with SP than in women with MP. In linear regression, preeclampsia was significantly associated with TSH (−0.675 IU/ml, *P* = 0.009), free T3 (0.977 pg/ml, *P* < 0.001), and free T4 (0.186 ng/dl, *P* < 0.001) levels. In contrast to anti-TG antibodies and TSH, Sudanese patients with preeclampsia had higher levels of T3 and T4 hormones and anti-TPO antibodies irrespective of parity, gestational age, and hemoglobin levels.

## Introduction

Preeclampsia is a multi-organ syndrome of pregnancy, characterized by the new onset of hypertension and proteinuria after 20 weeks of gestation (1). It is a big health problem where it is the main cause of maternal and perinatal morbidity and mortality worldwide (2). The exact underlying patho-physiology of preeclampsia is not yet fully understood; however, placental pathology leading to preeclampsia is suggested, with inadequate/inappropriate cytotrophoblast invasion, maternal endothelial dysfunction, and placental hypoxia as plausible explanations (3, 4).

Preeclampsia is associated with depressed thyroid functions (5). Recent reports showed that thyroid functions correlate with disease severity and obstetric outcomes in patients suffering from preeclampsia (6). Evaluation of thyroid function may therefore predict occurrence of preeclampsia (7). Moreover, there is accumulating evidence that possibility of preeclampsia increases if biochemical hypothyroidism coexists with certain thyroid autoantibodies (8, 9). Studies on the prevalence of anti-thyroid peroxidase (anti-TPO) and anti-thyroglobulin (anti-TG) antibodies indicate that thyroid autoimmunity significantly affects the well-being of the pregnant mother as well as the fetus independently of the thyroid dysfunction (10, 11).

In spite of the reports that recommend thyroid hormones [tri-iodothyronine (T3), thyroxine (T4), and thyroid-stimulating hormone (TSH)] and anti-TPO and anti-TG antibodies as reliable predictors of preeclampsia and obstetrical outcome, some investigators have concerns about the efficiency of these parameters (12–14). Controversy in this regard is probably justifiable by the inability of some studies to confirm associations between maternal thyroid function/antibodies and pregnancy/fetal complications (14–16).

There is an extremely high maternal mortality in Sudan where preeclampsia/eclampsia is the main cause of obstetric complications and maternal deaths (17, 18). In Sudan, several aspects of preeclampsia were investigated (19–23); however, there were no published data on thyroid function/antibodies in preeclamptic patients. This study aimed to investigate thyroid function (TSH, free T3, and free T4)/antibodies (anti-TPO and anti-TG) in patients with preeclampsia.

## Patients and Methods

A case–control study was conducted at Wad Medani Hospital, Central Sudan, during the period of March–July 2013. Cases were women with preeclampsia, which is defined as the occurrence of blood pressure ≥140 mm Hg systolic or ≥90 mm Hg diastolic arising after 20 weeks of gestation in a woman who is normotensive before 20 weeks plus presence of 300 mg or more of protein in a 24-h urine sample or ≥2+ in dipstick. Cases were further divided into mild preeclampsia (MP) and severe preeclampsia (SP) according to the diastolic blood pressure of <110 or ≥110 mm Hg, respectively. A consecutive control was taken for each case. Controls were normotensive women (without any blood pressure values >139/89 mm Hg or proteinuria) presented in the antenatal visits.

Women were excluded from the cases and controls if they had twins or had previous history of thyroid disease, hypertension, renal disease, diabetes, liver disease, or on medication that might affect thyroid function.

After signing an informed consent, the sociodemographic, medical history was taken from each woman (cases and controls) using a questionnaire. Blood pressure was measured using a sphygmomanometer for both case and control groups. Maternal weight and height were measured, and body mass index (BMI) was calculated and expressed as weight (kg)/height (m)^2^.

Then, 5 ml of venous blood was taken from each respondent, allowed to clot, centrifuged, and stored at −20°C using immunoassay analyzer (AIA 360, Tosoh, Japan), following the manufacturer’s instructions, till the assay of the thyroid hormones (TSH, free T3, and free T4). Specific anti-TPO and anti-TG antibody profiles were analyzed using commercial ELISA (Euroimmun, Lübeck, Germany) Kits.

A total sample size of 55 women in each arm of the study was calculated using a formula for the difference in the mean of the proposed variables (TSH, free T3, and free T4 and anti-TPO and anti-TG antibodies) which would provide 80% power to detect a 5% difference at α = 0.05 and assumed that 10% of women would not respond.

Because there were no published data on the normal value for TSH, free T3, and free T4 among pregnant Sudanese women, 2.5 and 97.5% of the controls were considered as referral values for these parameters in the third trimester.

### Statistical analysis

Data were entered in a computer using SPSS for Windows (version 16.0). Continuous data were compared between the groups (controls, MP, and SP) using ANOVA and Kruskal–Wallis *H* (Mann–Whitney *U* test between two groups) tests when the data were normally and not normally distributed, respectively. Multiple linear regression models were performed where TSH, Free T3, Free T4, anti-TPO, and anti-TG levels were continuous dependent variables and age, parity, gestational age, BMI index, and preeclampsia were the independent predictors of interest. A *P* value of <0.05 was considered to be significant.

### Ethics

Ethical clearance was obtained from the Research Board at the Faculty of Medicine, University of Khartoum.

## Results

The basic characteristics of the three groups of the controls, MP, and SP (55 women in each arm) are shown in Table 1 where there was no significant difference in the age, parity, and BMI. However, gestational age and hemoglobin were significantly lower in women with SP (Table 1).

**Table 1:** **Comparing the mean (SD)^a^ of the basic characteristics of women with preeclampsia and controls**.

Variable	Controls (*n* = 55)	Mild preeclampsia (*n* = 55)	Severe preeclampsia (*n* = 55)	*P*
Age (years)	27.4 (5.1)	28.1 (4.9)	29.1 (4.7)	0.217
Parity	2.2 (1.5)	2.7 (1.6)	2.9 (1.8)	0.065
Gestational age (weeks)	38.4 (2.0)	37.7 (1.3)	36.1 (2.9)	<0.001
Body mass index (kg/cm^2^)	24.3 (1.9)	24.2 (1.9)	24.6 (2.3)	0.568
Hemoglobin (g/dl)	11.2 (1.2)	10.3 (1.8)	10.0 (1.4)	<0.001

*^a^ANOVA test was used*.

**Figure 1:**
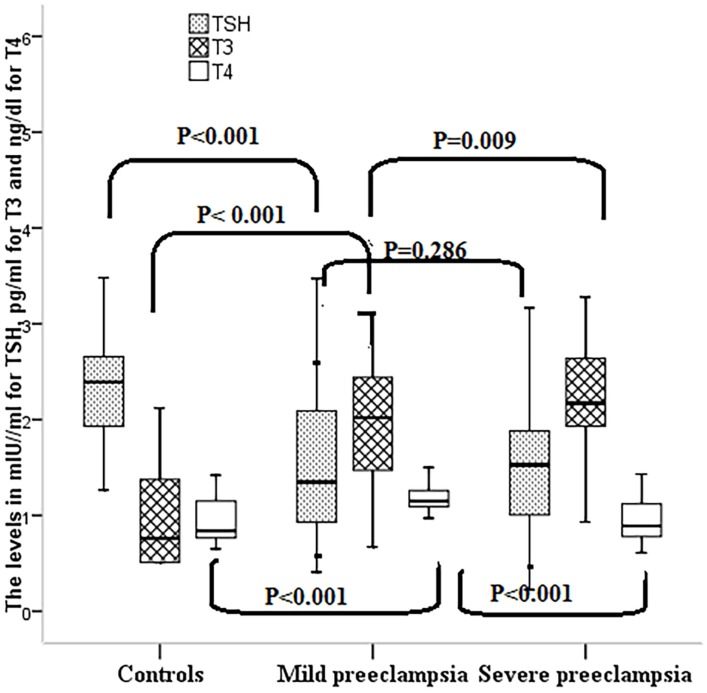
**TSH, free T3, and T4 among women with preeclampsia in Medani Hospital, Sudan**.

While the median (interquartile range) of TSH was significantly lower, both free T3 and free T4 levels were significantly higher in women with preeclampsia than in the healthy controls (HCs). There was no significant difference in the TSH levels in women with MP and SP (Table 2; Figure 1).

**Table 2:** **The median (interquartile) of thyroid function and antibodies in women with mild preeclampsia (MP), severe preeclampsia (SP), and healthy controls (HCs)**.

Variable	Healthy controls (*n* = 55)	Mild preeclampsia (*n* = 55)	Severe preeclampsia (*n* = 55)	*P*
TSH (mIU/ml)	2.3 (1.9–2.6)	1.3 (0.9–2.1)	1.5 (1.0–1.9)	HC vs. MP, *P* < 0.001
				HC vs. SP, *P* < 0.001
				MP vs. SP, *P* = 0.286
Free T3 (pg/ml)	0.7 (0.5–1.3)	2.0 (1.4–2.4)	2.1 (1.9–2.6)	HC vs. MP, *P* < 0.001
				HC vs. SP, *P* < 0.001
				MP vs. SP, *P* = 0.009
Free T4 (ng/dl)	0.8 (0.7–1.1)	1.1 (1.0–1.2)	0.9 (0.7–1.1)	HC vs. MP, *P* < 0.001
				HC vs. SP, *P* = 0.235
				MP vs. SP, *P* < 0.001
Anti-TPO (IU/ml)	8.3 (6.4–10.1)	8.7 (6.9–10.7)	10.5 (7.3–13.4)	HC vs. MP, *P* = 0.474
				HC vs. SP, *P* = 0.005
				MP vs. SP, *P* = 0.032
Anti-TG (IU/ml)	11.4 (8.5–13.8)	9.0 (6.8–11.0)	11.1 (8.2–13.2)	HC vs. MP, *P* = 0.002
				HC vs. SP, *P* = 0.820
				MP vs. SP, *P* = 0.008

In comparison with MP, women with SP had significantly higher levels of free T3 and significantly lower levels of free T4 (Table 2; Figure 1). In the control group, the 2.5% was 0.77 IU/ml, 0.51 pg/ml, and 0.65 ng/dl and 97.5% was 5.14 IU/ml, 3.00 pg/ml, and 1.38 ng/dl for TSH, free T3, and free T4, respectively. About 10 (9.0%), 0 (0%), and 1 (0.9%) of the 110 preeclamptic women had low levels of TSH, free T3, and free T4, respectively. On the other hand, 2 (1.8%), 4 (3.6%), and 5 (4.5%) of the 110 preeclamptic women had high TSH, free T3, and free T4, respectively.

**Figure 2:**
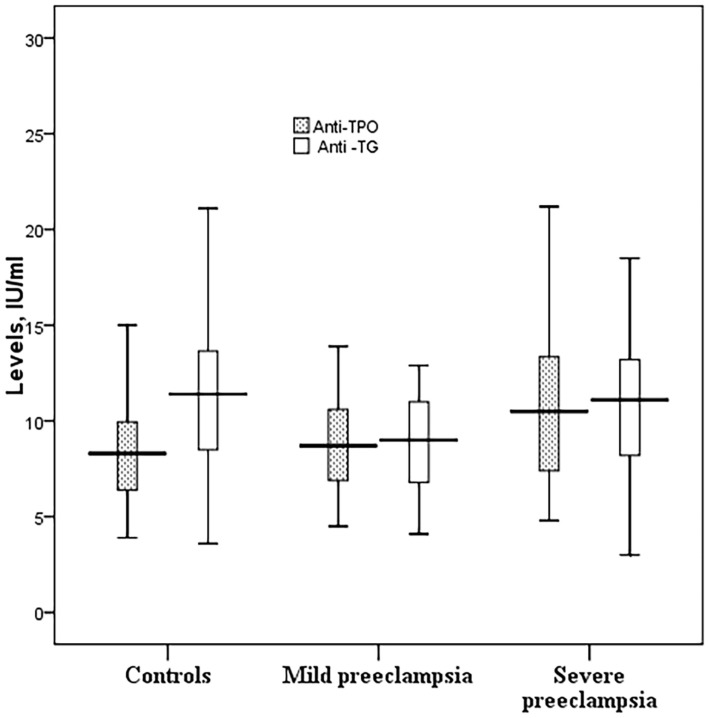
**Anti-TPO and anti-TG among women with preeclampsia in Medani Hospital, Sudan**.

While anti-TPO antibodies were significantly higher, anti-TG antibodies were significantly lower in women with preeclampsia. Likewise, anti-TPO antibodies were significantly higher and anti-TG antibodies were significantly lower in women with SP than in women with MP (Table 2; Figure 2).

In linear regression, preeclampsia was significantly associated with TSH (−0.675 IU/ml, *P* = 0.009), free T3 (0.977 pg/ml, *P* < 0.001), and free T4 (0.186 ng/dl, *P* < 0.001) levels (Table 3). Interestingly, there was no association between preeclampsia and both antibodies levels (Table 4).

**Table 3:** **Linear regression analysis of factors associated with TSH, Free T3, and Free T4 levels**.

Variable	TSH			Free T3			Free T4		
	*R* = 0.379 and *R*^2^ = 0.084			*R* = 0.683 and *R*^2^ = 0.429			*R* = 0.454 and *R*^2^ = 0.158		
	Coefficient	SE	*P*	Coefficient	SE	*P*	Coefficient	SE	*P*
Age	0.030	0.022	0.169	0.006	0.013	0.664	0.003	0.005	0.593
Parity	−0.032	0.064	0.620	−0.023	0.038	0.551	−0.014	0.014	0.313
Gestational age	−0.018	0.041	0.663	−0.011	0.022	0.605	0.016	0.008	0.061
Body mass index	−0.026	0.043	0.544	0.031	0.025	0.226	−0.006	0.009	0.547
Hemoglobin	0.001	0.057	0.980	−0.037	0.034	0.268	0.006	0.013	0.639
Preeclampsia	−0.675	0.253	0.009	0.977	0.126	<0.001	0.186	0.045	<0.001
Free T3	−0.183	0.139	0.192	–	–	–	0.513	0.215	0.018
Free T4	0.415	0.373	0.268	0.513	0.215	0.018	–	–	–
Anti-TPO	0.026	0.016	0.101	0.017	0.009	0.078	0.004	0.004	0.236
Anti-TG	−0.003	0.016	0.861	0.012	0.009	0.172	−0.011	0.003	0.001

**Table 4:** **Linear regression analysis of factors associated with anti-TPO and anti-TG levels**.

Variable	Anti-TPO			Anti-TG		
	*R* = 0.470 and *R*^2^ = 0.441			*R* = 0.179 and *R*^2^ = 0.151		
	Coefficient	SE	*P*	Coefficient	SE	*P*
Age	−0.156	0.108	0.151	0.100	0.127	0.433
Parity	0.746	0.317	0.020	−0.383	0.373	0.305
Gestational age	0.116	0.184	0.530	−0.288	0.217	0.185
Body mass index	0.118	0.210	0.577	0.070	0.248	0.778
Hemoglobin	0.037	0.289	0.898	0.168	0.341	0.623
Preeclampsia	−1.832	0.996	0.068	1.003	1.174	0.394
Anti-TG	0.397	0.068	<0.001	–	–	–

There was no association between TSH and free T3, free T4 and antibodies. Free T3 was significantly associated with free T4 and with anti-TPO and anti-TG. Free T4 was significantly associated with anti-TG (Table 3).

## Discussion

The main findings of the current study were then compared with HC women; preeclamptic women had significantly lower levels of TSH and anti-TG and significantly higher levels of free T3, free T4, and anti-TPO.

This finding disagrees with previous findings from other studies (24–26), although the reported deviations of free T3, free T4, and TSH concentrations from normal physiological levels were quite variable. A recent study showed lower thyroid hormones and higher TSH levels in both early and late preeclampsia groups compared to the control subjects; however, all hormone concentrations were comparable in both early and later preeclampsia (27). Zhou et al. observed that the decline in thyroid function is proportional to the deterioration in renal function that complicates cases of SP (8). A case–control study conducted in the antenatal clinic of a public hospital in Delhi showed significantly higher TSH levels in preeclampsia group compared to controls; however, mean values of thyroid hormones were in the normal range (25). In another study, maternal serum TSH was proven to be an acceptable predictor of premature labor in patients with preeclampsia (26). Alternatively, studies that failed to demonstrate hypothyroidism in patients with preeclampsia are not uncommon (11, 12, 14). Based on the results of Qublan et al., there were no significant differences in the levels of FT4, FT3, and TSH between the patients with SP and healthy normotensive controls regardless of the gestational age (15). Nine years later, Khadem and his group were able to reproduce Qublan et al.’s findings in Iranian pregnant women (12). Yet, at least one study reported a significantly higher T3 level in patients with SP compared to healthy pregnant women (13). This disagreement is possibly due to examining patients with different ethnicity, BMI, gestational ages, and parities (28–31).

In the current study, while anti-TPO antibodies were significantly higher, anti-TG antibodies were significantly lower in women with preeclampsia. Likewise, anti-TPO antibodies were significantly higher and anti-TG antibodies were significantly lower in women with SP than in women with MP. These findings agree with several previous reports (9, 32), but not others (14, 16). The study by Mecacci et al. reported positive anti-TPO in 33.3% of patients with preeclampsia, which was a significantly higher ratio when compared to the HC pregnant women (14.5%) (32). Contrary to this, a study evaluating the association between maternal thyroid dysfunction/antibodies and pregnancy complications failed to prove predictive values of these parameters for neither preeclampsia nor maternal mortality (16). A separate study adds more to the disputes by confirming higher levels of anti-TPO antibodies in HC subjects compared to patients with gestational hypertension, preeclampsia, or eclampsia (14).

Incongruent with previous reports, the present results fail to demonstrate influences of parity and gestational age on maternal thyroid function/antibodies (28, 33–35). A cross-sectional study conducted in 50 non-pregnant women as a control group, 50 primiparae and 50 multiparae women showed increased serum levels of T4 and T3 in both groups, while serum level of TSH decreased significantly in the two groups (35). Higher levels of thyroid hormones in multiparae women in iodine deficient regions may explain their higher risk to develop multinodular goiter (35). Based on the data used to establish pregnancy-specific thyroid function reference intervals of a southern Chinese population, TSH is likely to increase while thyroid hormones decrease in advanced gestational ages (28). The same findings were reproduced by Khalid and his group when they studied thyroid function in pregnant women attending a large, tertiary referral maternity hospital in Ireland (33). In addition, the study by Khalid et al. documented significant positive correlation between anti-TPO antibodies, gestational age and TSH, but not T4.

The limitations of the current study were that iodine was not investigated in these women and the thyroid function/antibodies were investigated in the third trimester after the diagnosis of the preeclampsia. There were no referral values for thyroid functions in pregnant Sudanese women. Yet, Elnagar et al. in a longitudinal small sample size study (28 women) reported that the mean FT4 values in Sudanese showed no significant changes during pregnancy, and 23, 40, and 14% of whom had FT4 values below the reference range in the first, second, and third trimesters, respectively, and none of them showed FT4 values above the reference range (36). The later study reported that these pregnant Sudanese women had iodine deficiency. Previous reports showed that the mean FT4 levels of the goitrous pregnant Sudanese women were significantly lower than that of the non-pregnant control group (37). A longitudinal study investigating the thyroid function/antibodies and iodine correlated with pregnancy outcome is needed.

## Conflict of Interest Statement

The authors declare that the research was conducted in the absence of any commercial or financial relationships that could be construed as a potential conflict of interest.
